# Platelet distribution width correlates with prognosis of gastric cancer

**DOI:** 10.18632/oncotarget.15561

**Published:** 2017-02-21

**Authors:** Xin Zhang, Ming-Ming Cui, Shuang Fu, Lu-Lu Li, Yan-Song Liu, Zhi-Ping Liu, Tiemin Liu, Rui-Tao Wang, Kai-Jiang Yu

**Affiliations:** ^1^ Department of Internal Medicine, The Third Affiliated Hospital, Harbin Medical University, Harbin, Heilongjiang, 150081, China; ^2^ Department of Geriatrics, The Second Affiliated Hospital, Harbin Medical University, Harbin, Heilongjiang, 150086, China; ^3^ Department of Intensive Care Unit, The Third Affiliated Hospital, Harbin Medical University, Harbin, Heilongjiang, 150081, China; ^4^ Department of Internal Medicine, University of Texas Southwestern Medical Center, Dallas, TX, 75390, USA; ^5^ Division of Hypothalamic Research, Department of Internal Medicine, UT Southwestern Medical Center, Dallas, TX, 75390, USA

**Keywords:** gastric cancer, platelet distribution width, prognosis, survival

## Abstract

**Background:**

Activated platelets promote tumor cell growth, aberrant angiogenesis, and invasion. However, the value of platelet indices for predicting survival in gastric cancer remains unknown. The goal of this study was to investigate the predictive significance of platelet indices in gastric cancer.

**Result:**

Reduced platelet distribution width (PDW) was significantly correlated with age, carcinoembryonic antigen, tumor stage, nodule stage, and tumor-nodule-metastases stage. Moreover, decreased PDW correlated with a shorter overall survival in gastric cancer. Multivariate analysis identified PDW as an independent prognostic factor for overall survival (hazard ratio: 0.493, 95% confidence interval: 0.319-0.761, p = 0.001).

**Method:**

A total of 294 patients with gastric cancer were retrospectively analyzed between January 2009 and December 2009. The association between platelet indices and overall survival were evaluated. The prognostic analysis was carried out with Cox regression model.

**Conclusion:**

PDW is easily available with routine blood counts. Our data revealed that reduced PDW is unfavorable prognostic factor in gastric cancer. Further studies are warranted.

## INTRODUCTION

Gastric cancer (GC) is the second leading cause of cancer-related mortality worldwide [1]. Although much progress has been made in the diagnosis and treatment of GC in recent years, the rate of diagnosis in early stage is still low and the prognosis of GC remains poor [2, 3]. Therefore, identification of new useful biomarkers for prognosis in patients with GC is of great importance.

Recent studies have demonstrated a significant role of platelets during cancer progression and metastases. Activated platelets promote tumor cell growth, aberrant angiogenesis, and invasion [4]. Elevated platelets are associated with a poor prognosis in various types of cancer, including pancreatic cancer, gastric cancer, colorectal cancer, endometrial cancer, and ovarian cancer [5–9]. However, total platelet count is determined by the balance between the rate of production and consumption of platelets. A normal platelet count could conceal the presence of highly hypercoagulative and pro-inflammatory cancer phenotypes in the presence of efficient compensatory mechanisms [10].

Mean platelet volume (MPV) is an index of activated platelets and is linked to different inflammatory conditions [11]. Platelet distribution width (PDW), another platelet parameter, indicates variation in platelet size and differentially diagnoses thrombocytopenia [12]. Additionally, both MPV and PDW are easily detected with routinely used hemocytometers. Recent studies reported that MPV is a biomarker in early diagnosis for GC and predicts chemotherapy response and prognosis in patients with unresectable gastric cancer [13, 14]. However, PDW has not been studied completely.

The purpose of this study was to investigate the prognostic impact of the preoperative platelet indices on the overall survival in patients with gastric cancer.

## RESULTS

The characteristics of the patients are summarized in Table 1. Overall, there were 206 (70.1%) male patients and 88 (29.9%) female patients, and the median age was 56.0 ± 10.6 years (range 24-81). In terms of the staging system, 40 cases were categorized as stage I, 88 as stage II, 148 as stage III and 18 as stage IV.

**Table 1:** Baseline characteristics of the patients according to the PDW

Variables	Totaln (%)	PDW ≤ 16.8n (%)	PDW > 16.8n (%)	P value
Age (years)				0.036
≤60	197 (67.0)	48 (57.8)	149 (70.6)	
>60	97 (33.0)	35 (42.2)	62 (29.4)	
Gender				0.602
Male	206 (70.1)	60 (72.3)	146 (69.2)	
Female	88 (29.9)	23 (27.7)	65 (30.8)	
T stage				< 0.001
T1	28 (9.5)	4 (4.8)	24 (11.4)	
T2	58 (19.7)	18 (21.7)	40 (18.9)	
T3	84 (28.6)	27 (32.5)	57 (27.0)	
T4	124 (42.2)	34 (41.0)	90 (42.7)	
N stage				0.013
N0	79 (26.9)	18 (21.7)	61 (28.9)	
N1	54 (18.4)	12 (14.4)	42 (19.9)	
N2	74 (25.2)	20 (24.1)	54 (25.6)	
N3	87 (29.6)	33 (39.8)	54 (25.6)	
Cancer Stage				< 0.001
I	40 (13.6)	10 (12.0)	30 (14.2)	
II	88 (29.9)	22 (26.5)	66 (31.3)	
III	148 (50.3)	40 (48.2)	108 (51.2)	
IV	18 (6.1)	11 (13.3)	7 (3.3)	
Tumor Size				0.360
≥ 5cm	88 (29.9)	31 (37.3)	67 (31.8)	
< 5cm	206 (70.1)	52 (62.7)	144 (68.2)	
Histology differentiation				0.801
Well/moderately	54 (18.4)	16 (19.3)	38 (18.0)	
Poorly	240 (81.6)	67 (80.7)	173 (82.0)	
CEA (ng/ml)				0.009
< 5	240 (81.6)	60 (72.3)	180 (85.3)	
≥ 5	54 (18.4)	23 (27.7)	31 (14.7)	
NLR				0.080
≤ 2.39	186 (63.3)	46 (55.4)	140 (66.4)	
> 2.39	108 (36.7)	37 (44.6)	71 (33.6)	
PLR				0.082
≤ 176.6	196 (66.7)	49 (59.0)	147 (69.7)	
> 176.6	98 (33.3)	34 (41.0)	64 (30.3)	

PDW, platelet distribution width; CEA, carcinoembryonic antigen; NLR, neutrophil-to-lymphocyte ratio; PLR, platelet-to-lymphocyte ratio.

The median value of PDW was 17.3% (range, 10.3-23.2). ROC analysis showed that the optimal cutoff value for the PDW was 16.8 for the OS. The specificity and sensitivity were 42.6%, 78.5%, respectively (AUC = 0.590, 95% CI: 0.532-0.647, p = 0.015). According to the cutoff level, patients were divided into two groups. Of the total of 294 patients, 83 patients (28.2%) were detected with PDW of less than or equal to 16.8, while there were 211 patients (71.8%) whose PDW was greater than 16.8. Correlations between the PDW and clinicopathologic parameters are shown in Table 2. There were no significant differences in age (continuous variable), gender, WBC, NLR (categorical variable), PLR (categorical variable), lymphocytes, tumor size, and differentiation between the two groups. However, age (categorical variable), FPG, hemoglobin, neutrophils, platelet count, MPV, NLR (continuous variable), PLR (continuous variable), CEA, T stage, N stage, and TNM stage in two groups show significant differences.

**Table 2:** Baseline characteristics of the patients according to the PDW

Variables	PDW ≤ 16.8	PDW > 16.8	P value
Age (years)	57.3 (10.8)	55.5 (10.5)	0.204
FPG (mmol/L)	5.20 (4.80-5.60)	5.00 (4.55-5.50)	0.018
WBC (×109/L)	6.85 (2.84)	6.20 (1.71)	0.056
Neutrophils (×109/L)	4.49 (2.53)	3.74 (1.47)	0.012
Lymphocytes (×109/L)	1.81 (0.80)	1.85 (0.66)	0.655
Hemoglobin (g/dl)	122.8 (30.1)	130.2 (26.2)	0.036
Platelet count (×109/L)	294.6 (109.0)	260.8 (84.6)	0.012
MPV (fL)	8.2 (1.1)	8.9 (1.5)	<0.001
NLR	2.91 (2.15)	2.32 (1.51)	0.025
PLR	185.5 (89.5)	158.6 (86.2)	0.018

Data are expressed as means (SD) or median (IQR). FPG, fasting plasma glucose; WBC, white blood cell; MPV, mean platelet volume. Abbreviations: see Table 1.

**Figure 1:**
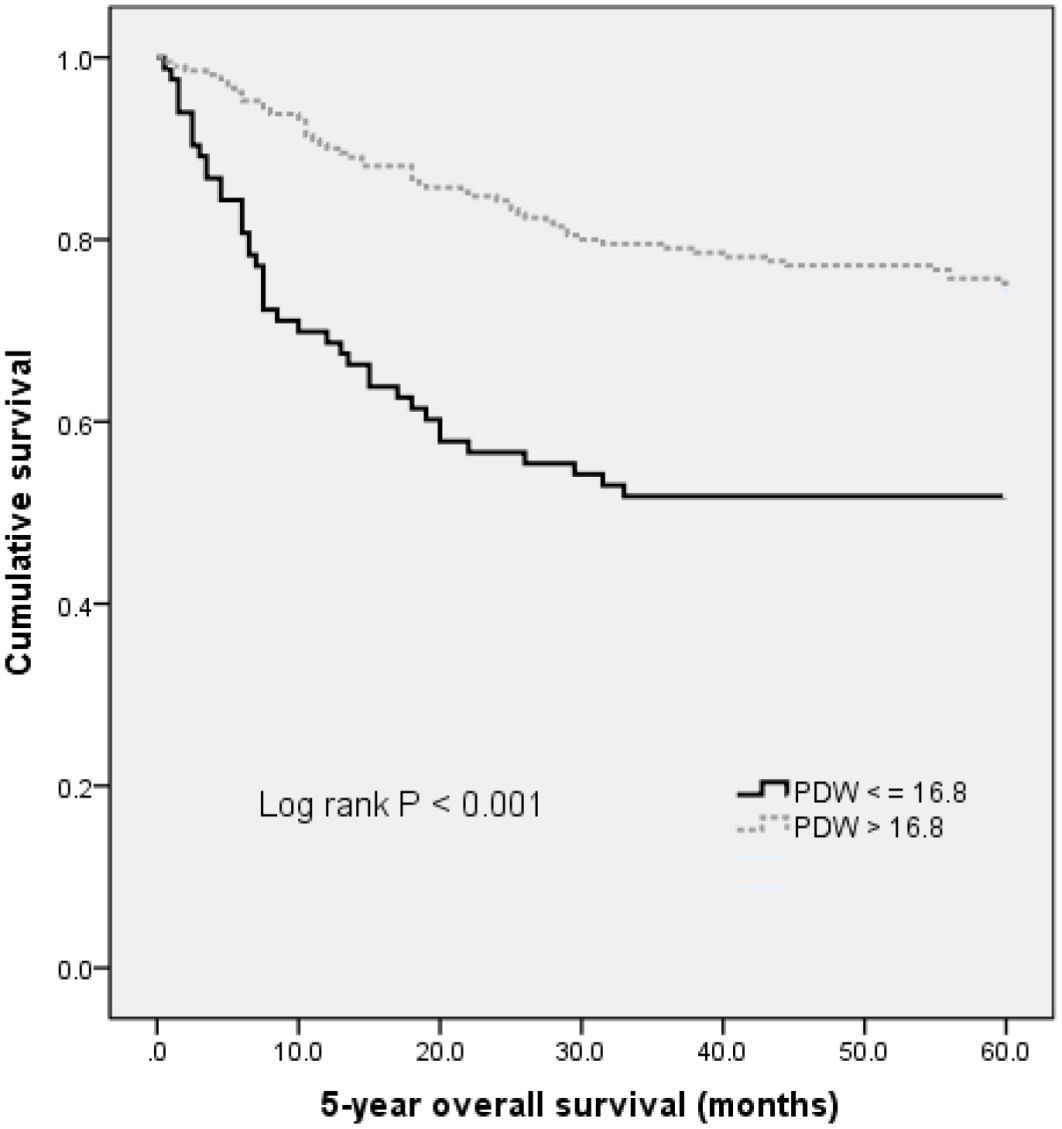
Kaplan–Meier analysis of overall survival in gastric cancer patients

With a median follow up of 60 months, 94 (32.0%) patients had death events. Patients with PDW less than or equal to 16.8 showed a shorter OS than patients with PDW of greater than 16.8 (17.2 *vs*. 59.7 months, p < 0.001). The Kaplan-Meier OS curves of the normal versus elevated PDW showed a significant separation (Figure 1).

In univariate analysis, age (categorical variable), T stage, N stage, TNM stage, histology differentiation, CEA, WBC, PDW, NLR and PLR were all associated with OS (see Table 3). Other parameters were not found to be in correlation with OS. Next, variables that showed a p value < 0.10 in univariate analysis were included in the multivariate analysis (see Table 4). Age, TNM stage, CEA, histology differentiation and PDW were independent prognostic markers for OS. Notably, PDW before treatment was an independent factor for OS, with HR of 0.493 (95% CI: 0.319-0.761, p = 0.001).

**Table 3:** Result of the univariate analysis of overall survival in patients with gastric cancer

	Hazard ratio	95% CI	*P*-value
Age (years) (> 60 versus ≤ 60)	1.803	1.200–2.709	0.005
Gender (male versus female)	1.031	0.660–1.612	0.893
FPG (mmol/L)	1.410	0.300–6.616	0.663
T stage	1.267	1.020–1.574	0.032
N stage	1.334	1.115–1.595	0.002
Cancer Stage(II+III+IV versus I)	2.281	1.459–3.568	< 0.001
Tumor Size (cm) (> 5 versus ≤ 5)	1.135	0.736–1.751	0.567
Histology differentiation(well/moderately versus poorly)	2.098	1.089–4.043	0.027
CEA (ng/ml) (> 5 versus ≤ 5)	2.211	1.414–3.459	0.001
WBC (×109/L)	1.141	1.045–1.246	0.003
Hemoglobin (g/dl)	0.995	0.988–1.003	0.204
Platelet count (×109/L)	1.000	0.999–1.001	0.611
MPV (fL)	1.095	0.962–1.246	0.168
PDW (%) (> 16.8 versus ≤ 16.8)	0.413	0.274–0.621	<0.001
NLR (> 2.39 versus ≤ 2.39)	2.281	1.521–3.421	<0.001
PLR (> 176.6 versus ≤ 176.6)	2.313	1.543–3.468	<0.001

Abbreviations: see Table 1 and Table 2.

**Table 4:** Result of the multivariate analysis of overall survival in patients with gastric cancer

	Hazard ratio	95% CI	*P*-value
Age (years) (> 60 versus ≤ 60)	1.649	1.090–2.496	**0.018**
T stage	0.964	0.742–1.252	0.782
N stage	1.036	0.847–1.266	0.731
Cancer Stage(II+III+IV versus I)	3.730	1.023–13.603	**0.046**
Histology differentiation(poorly versus well/moderately)	2.252	1.127–4.499	**0.021**
CEA (ng/ml) (> 5 versus ≤ 5)	1.664	1.035–2.673	**0.035**
WBC (×109/L)	1.074	0.980–1.178	0.128
PDW (%) (> 16.8 versus ≤ 16.8)	0.493	0.319–0.761	**0.001**
NLR (> 2.39 versus ≤ 2.39)	1.387	0.841–2.288	0.200
PLR (> 176.6 versus ≤ 176.6)	1.527	0.951–2.451	0.080

Variables that showed a p-value < 0.10 in univariate analysis were included in a multivariate Cox proportional hazards regression model using a backward elimination strategy. CI, confidence interval. Abbreviations: see Table 1 and Table 2

## DISCUSSION

This study showed that PDW is correlated with patient's survival and that PDW is an independent risk factor for prognosis.

Despite best current medical and surgical treatment, the overall prognosis of patients with gastric cancer remains poor. Numerous studies point to the key roles of platelet activation in tumor progression. Thrombocytosis is linked to reduced survival in patients with various tumor types, including lung cancer, ovary cancer, endometrium cancer, rectum cancer, kidney cancer, stomach cancer, pancreas cancer, brain cancer, and breast cancer. In gastric cancer, platelet-derived growth factor (PDGF) beta-receptor expression significantly correlates with less favorable clinicopathological parameters and shorter survival [15]. Moreover, platelet-derived growth factor-D contributes to aggressiveness of gastric cancer cells by up-regulating Notch and NF-κB signaling pathways [16]. Consistent to previous findings, our study indirectly confirmed the results using a simple parameter of platelet activation. These data are also in line with the current knowledge that anti-platelet is considered to be a part of cancer adjuvant therapy [4].

However, the specific mechanism by which PDW affect cancer progression is unclear. Bone marrow cells (including megakaryocytes) malfunction may be related to the decreased PDW. PDW is a measure of platelet heterogeneity caused by heterogeneous demarcation of megakarocytes [17]. Megakaryocytic maturation, platelet production and platelet size could be regulated by cytokines, such as interleukin-6 (IL-6), granulocytes colony stimulating factor (G-CSF) and macrophage colony stimulating factor (M-CSF) [18]. IL-6 promotes tumor angiogenesis, metastasis and metabolism [19]. Moreover, the cytokines G-CSF and M-CSF that be secreted by tumor cells could stimulate megakaryopoiesis and subsequent thrombopoiesis in cancer [20]. However, the clinical value of PDW has not been reported in gastric cancer. Another possible mechanism is that platelets promote the hypercoagulable state in cancer. Activated platelets create a procoagulant micro-environment that enables the tumor cells to cover themselves with platelets and evade the host immune system. High platelet-derived growth factor (PDGF)-D expression is strongly associated with tumor recurrence, distant metastasis and poor outcomes in gastric cancer [21]. Furthermore, PDGF-B is involved in the maintenance of microvessels in gastric cancer [22]. In addition, PDGF-A contributes to the development of pulmonary tumor thrombotic microangiopathy [23]. Those findings are consistent with the idea that activated platelets are involved the pathogenesis of gastric cancer.

Our study has a number of limitations that deserve mention. First, this was a single-center retrospective study and multicentric prospective studies are needed to reduce selection bias. Second, underlying mechanism is needed to support the potential application of PDW in gastric cancer. Third, the participants were composed of Chinese. Our results cannot thus be extrapolated to other ethnic groups.

In conclusion, PDW is easily available with routine blood counts. Our data revealed that reduced PDW is unfavorable prognostic factor in gastric cancer. Further studies are warranted to clarify the exact role of PDW in gastric cancer.

## PATIENTS AND METHODS

### Study population

This study included 294 gastric cancer cases treated at the Third Affiliated Hospital, Harbin Medical University from January 2009 and December 2009. Cases were included if they fulfilled the following criteria: (1) undergone complete surgical resection and diagnosis of gastric cancer was confirmed by histology; (2) without distant metastasis at diagnosis; (3) untreated before diagnosis. Exclusion criteria included: hematological disorders, autoimmune diseases, systemic inflammatory diseases, coronary artery disease, hypertension, diabetes mellitus, thyroid disease, renal disease, hepatic disorder and other cancer, and medical treatment with anticoagulant, statins, and acetylic salicylic acid.

The date of surgery was regarded as the starting point of the survival follow-up until December 31, 2014. Overall survival (OS) was defined as the period from surgery to death or the last follow-up. The median follow-up duration was 60 months.

Written informed consents were obtained from all patients. This study was approved by the Institutional Review Board of the 3rd Affiliated Hospital of Harbin Medical University.

### Clinical examination and biochemical measurements

All the subjects underwent physical examination. Body mass index (BMI) was calculated as the ratio of weight (kg) to height squared (m^2^). Clinical data including smoking status, medical history and medication use were recorded for each subject. Venous blood samples after an 10-hour overnight fasting were collected from the individuals within 1 week prior to surgery. White blood cell (WBC), haemoglobin, and platelet indices were measured by an autoanalyzer (Sysmex XE-2100, Kobe, Japan). The whole blood samples were collected in EDTA-containing tubes, and all samples were processed within 30 minutes after blood collection. The inter- and intra-assays coefficients of variation (CVs) of all these assays were below 5%.

The platelet-to-lymphocyte ratio (PLR) was calculated as the absolute platelet count measured in × 10^9^/L divided by the absolute lymphocyte count measured in ×10^9^/L. The neutrophil-to-lymphocyte ratio (NLR) was calculated as the absolute neutrophil count measured in × 10^9^/L divided by the absolute lymphocyte count measured in × 10^9^/L. The ideal cutoff values for PDW, PLR, and NLR were determined applying receiver operating curve analysis (see Supplementary Figure 1).

### Statistical analysis

The descriptive statistics are presented as means ± SD or medians (interquartile range) for continuous variables and percentages of the number for categorical variables. When baseline characteristics between two groups were compared, normally distributed continuous variables were compared with the Student t test and skewed-distributed with the Mann-Whitney U test. When baseline characteristics among three groups were compared, normally distributed continuous variables were compared with the one-way ANOVA and skewed-distributed with Kruskal-Wallis H test. The Chi-square test was used for categorical variables. The Kaplan-Meier method and the log-rank test were used for the comparison of survival rates. Variables that showed a p value < 0.1 in univariate analysis were included in a multivariate Cox proportional hazards regression model using a backward elimination strategy. Receiver-operating characteristics (ROC) curve analysis was performed to identify cut-off value of PDW. A two-tailed p value less than 0.05 was considered significant in all tests. All statistical analyses were performed using SPSS Statistics version 22.0 (SPSS Inc., Chicago, IL, USA).

## SUPPLEMENTARY MATERIALS FIGURES



## References

[1] Alexandrov LB, Nik-Zainal S, Siu HC, Leung SY, Stratton MR (2015). A mutational signature in gastric cancer suggests therapeutic strategies. Nat Commun.

[2] Wang Z, Liu H, Shen Z, Wang X, Zhang H, Qin J, Xu J, Sun Y, Qin X (2015). The prognostic value of CXC-chemokine receptor 2 (CXCR2) in gastric cancer patients. BMC Cancer.

[3] Fukuda Y, Yamamoto K, Hirao M, Nishikawa K, Nagatsuma Y, Nakayama T, Tanikawa S, Maeda S, Uemura M, Miyake M, Hama N, Miyamoto A, Ikeda M (2016). Sarcopenia is associated with severe postoperative complications in elderly gastric cancer patients undergoing gastrectomy. Gastric Cancer.

[4] Mezouar S, Frere C, Darbousset R, Mege D, Crescence L, Dignat-George F, Panicot-Dubois L, Dubois C (2016). Role of platelets in cancer and cancer-associated thrombosis: Experimental and clinical evidences. Thromb Res.

[5] Suzuki K, Aiura K, Kitagou M, Hoshimoto S, Takahashi S, Ueda M, Kitajima M (2004). Platelets counts closely correlate with the disease-free survival interval of pancreatic cancer patients. Hepatogastroenterology.

[6] Long Y, Wang T, Gao Q, Zhou C (2016). Prognostic significance of pretreatment elevated platelet count in patients with colorectal cancer: a meta-analysis. Oncotarget.

[7] Pietrzyk L, Plewa Z, Denisow-Pietrzyk M, Zebrowski R, Torres K (2016). Diagnostic Power of Blood Parameters as Screening Markers in Gastric Cancer Patients. Asian Pac J Cancer Prev.

[8] Ekici H, Malatyalioglu E, Kokcu A, Kurtoglu E, Tosun M, Celik H (2015). Do Leukocyte and Platelet Counts Have Benefit for \Preoperative Evaluation of Endometrial Cancer. Asian Pac J Cancer Prev.

[9] Qiu J, Yu Y, Fu Y, Ye F, Xie X, Lu W (2012). Preoperative plasma fibrinogen, platelet count and prognosis in epithelial ovarian cancer. J Obstet Gynaecol Res.

[10] Seretis C, Youssef H, Chapman M (2015). Hypercoagulation in colorectal cancer: what can platelet indices tell us. Platelets.

[11] Gasparyan AY, Ayvazyan L, Mikhailidis DP, Kitas GD (2011). Mean platelet volume: a link between thrombosis and inflammation. Curr Pharm Des.

[12] Kaito K, Otsubo H, Usui N, Yoshida M, Tanno J, Kurihara E, Matsumoto K, Hirata R, Domitsu K, Kobayashi M (2005). Platelet size deviation width, platelet large cell ratio, and mean platelet volume have sufficient sensitivity and specificity in the diagnosis of immune thrombocytopenia. Br J Haematol.

[13] Kilincalp S, Ekiz F, Basar O, Ayte MR, Coban S, Yılmaz B, Altınbaş A, Başar N, Aktaş B, Tuna Y, Erbiş H, Uçar E, Erarslan E (2014). Mean platelet volume could be possible biomarker in early diagnosis and monitoring of gastric cancer. Platelets.

[14] Lian L, Xia YY, Zhou C, Shen XM, Li XL, Han SG, Zheng Y, Gong FR, Tao M, Li W (2015). Mean platelet volume predicts chemotherapy response and prognosis in patients with unresectable gastric cancer. Oncol Lett.

[15] Paulsson J, Sjoblom T, Micke P, Pontén F, Landberg G, Heldin CH, Bergh J, Brennan DJ, Jirström K, Ostman A (2009). Prognostic significance of stromal platelet-derived growth factor beta-receptor expression in human breast cancer. Am J Pathol.

[16] Ahmad A, Wang Z, Kong D, Ali R, Ali S, Banerjee S, Sarkar FH (2011). Platelet-derived growth factor-D contributes to aggressiveness of breast cancer cells by up-regulating Notch and NF-kappaB signaling pathways. Breast Cancer Res Treat.

[17] Paulus JM (1981). Recent advances in the story of megakaryocyte physiology. Pathol Biol (Paris).

[18] Kaushansky K (1998). Growth factors and hematopoietic cell fate. A new feature: controversies in hematology. Blood.

[19] Kumari N, Dwarakanath BS, Das A, Bhatt AN Role of interleukin-6 in cancer progression and therapeutic resistance. Tumour Biol.

[20] Kowanetz M, Wu X, Lee J, Tan M, Hagenbeek T, Qu X, Yu L, Ross J, Korsisaari N, Cao T, Bou-Reslan H, Kallop D, Weimer R (2010). Granulocyte-colony stimulating factor promotes lung metastasis through mobilization of Ly6G+Ly6C+ granulocytes. Proc Natl Acad Sci U S A.

[21] Ogawa N, Inokuchi M, Takagi Y, Sugita H, Kato K, Kojima K, Sugihara K (2015). Clinical significance of platelet derived growth factor-C and -D in gastric cancer. Oncol Lett.

[22] Suzuki S, Dobashi Y, Hatakeyama Y, Tajiri R, Fujimura T, Heldin CH, Ooi A (2010). Clinicopathological significance of platelet-derived growth factor (PDGF)-B and vascular endothelial growth factor-A expression, PDGF receptor-beta phosphorylation, and microvessel density in gastric cancer. BMC Cancer.

[23] Abe H, Hino R, Fukayama M (2013). Platelet-derived growth factor-A and vascular endothelial growth factor-C contribute to the development of pulmonary tumor thrombotic microangiopathy in gastric cancer. Virchows Arch.

