# Supplementary material for: Platelet distribution width correlates with prognosis of gastric cancer
Source: Oncotarget. 2017 Feb 21;8(12):20213–9. doi: 10.18632/oncotarget.15561 (PMC5386756; doi:10.18632/oncotarget.15561)
Supplement: Supplementary file 1 [file oncotarget-08-20213-s001.pdf]

Platelet distribution width correlates with prognosis of gastric cancer

SUPPLEMENTARY FIGURE

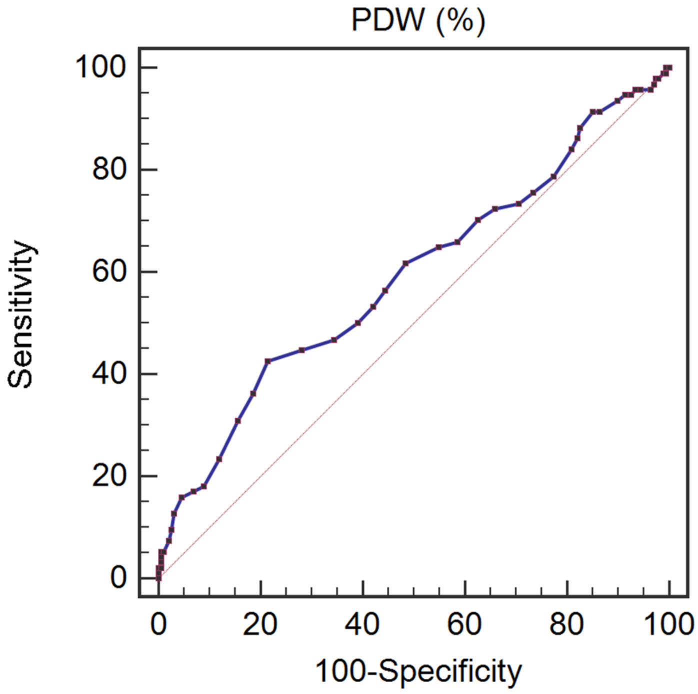

Supplementary Figure 1: Optimised cut-off was determined for PDW using standard ROC curve analysis.
